# Effect of language therapy alone for developmental language disorder in children: A meta-analysis

**DOI:** 10.3389/fpsyg.2022.922866

**Published:** 2022-10-03

**Authors:** Shengfu Fan, Bosen Ma, Xuan Song, Yuhong Wang

**Affiliations:** ^1^Department of Foreign Languages, Changzhi Medical College, Changzhi, China; ^2^School of International Studies, Zhejiang University, Hangzhou, China; ^3^Emergency Department, Dongying Honggang Hospital, Dongying, China

**Keywords:** randomized controlled trial, RevMan, long-term effect, linguistics, stratification

## Abstract

Despite numerous studies on the treatment of developmental language disorder (DLD), the intervention effect has long been debated. Systematic reviews of the effect of language therapy alone are rare. This evidence-based study investigated the effect of language therapy alone for different expressive and receptive language levels in children with DLD. Publications in databases including PubMed, the Cochrane Library, the Wanfang Database and the China National Knowledge Infrastructure were searched. Randomized controlled trials were selected. The methodological quality of the included trials was assessed using the modified Jadad method. RevMan 5.3 software was used for the data analysis. Fifteen trials were included in this study. Compared with the control (no or delayed intervention) group, the intervention group showed significant differences in overall expressive language development [standard mean differences (SMD), 0.46; 95% confidence interval (CI), 0.12–0.80], mean length of utterances in a language sample (SMD, 2.16; 95% CI, 0.39–3.93), number of utterances in a language sample (SMD, 0.52; 95% CI, 0.21–0.84), parent reports of expressive phrase complexity (SMD, 1.24; 95% CI, 0.78–1.70), overall expressive vocabulary development (SMD, 0.43; 95% CI, 0.17–0.69) and different words used in a language sample (SMD, 0.62; 95% CI, 0.35–0.88). However, language therapy did not show satisfactory long-term effects on DLD. Although language therapy is helpful in improving the performance of children with DLD, its long-term effect is unsatisfactory.

## Introduction

Language disorders in children may have various causes, such as general developmental and emotional difficulties, autism and neurological impairment, but there are conditions in which no known etiology can be used to explain its presence. Under such conditions, the delay is termed developmental language disorder (DLD) (Bioshop et al., 2017). For this outstanding characteristic of DLD, scholars have used multiple terms to refer to this condition, such as specific language impairment and primary language disorder (Law et al., 2004) (this term conveys information about the mysterious origin of the delay, that is, this condition is “primary” rather than “secondary”). Additionally, as one of the most noticeable manifestations of DLD is “language delays” at an early age, scholars have also used the term “developmental language delay” (Fan et al., 2021) for it. According to the literature (Tomblin et al., 1997), DLD affects ~7% of preschool children. DLD is more often found in males than in females, with a sex ratio of 1.2:1 (Mouridsen and Hauschild, 2010). Children with “language delays” may obtain great improvement in language acquisition or even “catch up” with reference norms with age (Sylvestre et al., 2018); however, this is not the case for DLD, as children who suffer from this condition exhibit persistent difficulties into middle childhood or even beyond (Bioshop et al., 2017), which negatively affect the quality of life of patients as well as families.

DLD can manifest in different forms [e.g., expressive (language production) vs. receptive (language perception and comprehension)] and at different linguistic levels (e.g., phonetics, phonology, vocabulary, syntax, and pragmatics). Targeting the mosaic of various types of DLDs, scholars and language therapists have made many valuable attempts. Girolametto et al. (1997) used focused lexical stimulation to intervene in the phonological development of 25 children with expressive vocabulary delays and found that these clients exhibited gains in the phonological domain. Emphasizing the role of parents in facilitating the language development of DLD children, Allen and Marshall (2011) found that those children receiving parent-child interaction therapy over a period of 4 weeks showed gains in multiple linguistics domains, such as verbal initiations, the mean length of utterances and the proportion of child-to-parent utterances. Smith-Lock et al. (2013) investigated the effectiveness of different degrees of grammar interventions for DLD children and concluded that a weekly frequency of intervention over 8 weeks helped clients obtain much more gains in expressive grammar than did a daily intervention over 8 days. However, the effectiveness of language intervention in treating DLD in children has long been a matter of debate (Bioshop et al., 2017), particularly in regard to long-term treatment efficacy (Almost and Rosenbaum, 1998; Ebbels et al., 2007; Hampton et al., 2017). To solve these controversies, evidence-based systematic reviews may help.

To date, a number of evidenced-based studies on language intervention have been released. Roberts and Kaiser (2011) conducted a meta-analysis to investigate the effects of parent-implemented interventions on the development of the language skills of children aged between 18 and 60 months, and according to them, parent-implemented language interventions are effective for young children with language impairments. However, their study focused on both primary and secondary language impairments. In 2004, Law et al. (2004) conducted a meta-analysis regarding the possible gains of children with DLD at different linguistic levels after intervention, and they did not find much evidence to support the efficacy of language intervention, except for the expressive phonological level. They also investigated the difference in effect between parent-guided intervention and clinician-guided intervention, and did not observe any significant differences between the two approaches. However, it is noteworthy that since 2004, no additional quantitative summaries have been conducted to summarize the possible gains of language intervention for children with DLD from the perspective of different linguistic levels. Considering that numerous studies regarding language intervention therapies for DLD might have been conducted since the publication of Law et al.'s study, an updated systematic review should be conducted. However, the methodology described in in Law et al.'s study is very difficult to follow due to lack of sufficient details. In addition, what Law et al.'s study focused on is the immediate effect of intervention upon children with DLD; to the best of our knowledge, no systematic review has examined the long-term effect of language intervention on DLD. Though a few systematic reviews were released in the literature in recent years, they focused either on the explorations of the optimal intervention dosage (Frizelle et al., 2021a) and dose form (Frizelle et al., 2021b) or on the effect of intervention in a particular linguistic domain (Ebbels, 2014; Cleave et al., 2015).

Based on the aforementioned information, we conducted the current meta-analysis to investigate the effect of language therapy alone for DLD in children. The following are the main questions we aimed to solve: (1) What could children with DLD gain from language intervention alone in terms of different linguistic levels? (2) Which intervention method could help them benefit more, the parent-directed approach or the therapist/therapist assistant/clinician (non-parent)-directed approach? (3) What would be the long-term effect of language intervention alone, like after some time interval from the completion of the intervention? The results of this study may deepen our understanding of DLD and provide useful data for future treatment of this condition.

## Materials and methods

### Registration

The current meta-analysis was not registered.

### Initial article identification

Considering wide coverage as well as accessibility, the databases searched in this study included PubMed, the Cochrane Library, PsycINFO, the Wanfang Database and the China National Knowledge Infrastructure (CNKI). Literature searches were performed in June 2021.

Lack of consensus about terminology for DLD greatly affects literature retrieval outcomes. To avoid mis-retrieval of qualified articles as well as to ensure broad article inclusion, the population, intervention, comparison, outcome and study design (PICOS) framework (Ghaffari et al., 2018) was simplified for use in this study. The search strategy was primarily designed for the PubMed database. We used (((speech delay) or (language delay) or (speech disorder) or (language disorder) or (speech impairment) or (language impairment)) AND ((children) or (childhood)) AND ((speech training) or (language training) or (speech therapy) or (language therapy) or (speech treatment) or (language treatment)) AND ((clinical randomized trial) or (clinical randomized case-control trial) or (clinical trial))) as free-text terms, and the article type was set at all types except for reviews and systemic reviews.” For the Cochrane Library database, the same strategy was used, except that we excluded Cochrane reviews and Cochrane protocols. For PsycINFO, we set limitations on the publications involving children aged from birth to 12 years, in line with the age grouping method employed in this database. For Wangfang and CNKI, we used the corresponding Chinese translations as the command terms, and the retrieval manner was set at “fuzzy.” There were no exclusions based on language.

The outcomes were defined as any result related to the expressive and receptive levels of phonology (e.g., consonants correct in conversation speech and average of phonological deviations), semantics (e.g., overall vocabulary), morphology and syntax (e.g., overall expressive and receptive language, mean length of utterances in a language sample, number of utterances in a language sample and grammar ability), pragmatics (e.g., conversation initiation and turn-taking) and suprasegments (e.g., rhythm and intonation) of the language system.

The searches identified 2,623 publications in total, which included 1,235 from PubMed, 832 from Cochrane, 338 from PsycINFO, 198 from Wanfang and 20 from the CNKI. These publications were then entered into Endnote (version X9) for management, and 2,167 publications were included for further identification, with 456 discarded due to duplications.

### Inclusion/exclusion criteria and further article identification

The 2,167 publications obtained were further included or excluded according to the following criteria:

1) The publication had to focus on DLD, and those describing language delay as a secondary condition or as a comorbidity of another pathological condition, such as visual impairment, congenital cleft palate, hearing impairment, mental disability (e.g., autism) and central nervous system impairment, were excluded.

2) Only randomized case-control trials were included, whereas those involving a self-control design would be excluded.

3) The diagnostic criteria for DLD needed to be clearly described in the publication. To exclude the possibility that some qualified publications might be missed, we used language levels measured to be at least one standard deviation lower than the mean according to standardized tests or <50 expressive words at the age of 2 years as our inclusion criterion, in accordance with that described in the literature (Roberts and Kaiser, 2011). The recruited clients had to be aged between 18 months (Roberts and Kaiser, 2011; Cleave et al., 2015) and 12 years (in line with the age grouping method used in PsycINFO as well as according to Bioshop et al., 2017), and they had to have no general developmental disorders which could be indicative of a language disorder, such as disorders involving cognition or intelligence.

4) The intervention described in the publication must be subject to routine language/speech therapy, and those involving medication or assistance with medical appliances or computer were excluded. The intervention should last no <8 weeks to maximize the effect of speech/language training (Law et al., 2004).

5) Studies involving bilinguals were excluded due to the consideration that “the silent period” (Tamiya, 2014) may make the conditions in toddlers more complicated.

6) To guarantee higher homogeneity among the included research, studies using continuous variables to describe the outcomes were included, whereas those merely resorting to categorical variables were excluded.

7) Only studies whose measurement data were available, either as Supplementary material or upon request by phone or email, were considered.

All included publication underwent two rounds of further identification. In the first round, the publications were preliminarily screened based on the title and abstract to exclude those irrelevant to the purpose of this study. The publications that remained were then subjected to the second round of identification through full-text reading in strict accordance with the inclusion and exclusion criteria. Both rounds of identification were performed independently by two investigators (SFF and XS). The screening outcomes were separately introduced into the Endnote software and then compared. When a disagreement occurred, a final judgment was made through discussion between the investigators.

### Outcome coding and data processing

The outcomes reported in the included publications were recoded according to different expressive and receptive linguistic levels, and this process was independently completed by two investigators of our team.

To ensure sufficient data for the meta-analyses at different levels, both primary and secondary outcomes of the same publication were considered, as the same primary variables in one publication might be the secondary variables in another. For a publication that concerns multiple intervention groups, data were pooled and then compared with those of the control group. For a publication that could not be analyzed due to a lack of source data, the corresponding author was contacted by email or phone; otherwise, it was discarded. For any linguistic level, the analysis was abandoned when only one publication was included.

### Quality assessment

The modified Jadad scale, which is calculated based on randomization, allocation concealment, blinding, and withdrawals and dropouts (Wu et al., 2017; Xu et al., 2018), was used to assess the quality of the clinical trials. A score between 0 and 3 points indicates a high risk of bias, a score between 4 and 5 indicates a moderate risk of bias, and that between 6 and 7 indicates a low risk.

All included studies were independently assessed for the risk of bias by the two investigators (SFF and XS). When a disagreement arose, the third investigator (BSM) would join the discussion till agreement was reached.

### Statistical analysis

Data were processed with SPSS 25.0 and RevMan 5.3. The continuous variables were presented as either standard mean differences (SMDs) or weighted mean differences (WMDs). The *I*^2^ test was used to calculate the heterogeneity of the trials and to determine the adoption of a random effects model or a fixed effects model. The total effect and 95% confidence interval (95% CI) were calculated. Publication biases were assessed using the funnel plot method. To compare the effects of parent-based intervention and nonparent-based intervention, stratification analysis was performed.

## Results

### Final sample of included publications

After the first round of identification based on the title and abstract, 1,931 articles were excluded in due to a focus on other conditions, such as autism spectrum disorder, stuttering and attention deficit [Cohen's kappa = 0.62; 95% CI (0.49–0.75)]. After further careful examination, 21 articles were found to satisfy the inclusion criteria, with 97 excluded due to a lack of interventions (mostly observation studies), 44 excluded as they belonged to the categories of literature reviews, case reports or protocols, 67 excluded due to bilingual involvement or a shorter intervention period than required in this study and 7 excluded due to the use of a qualitative method [inter-rater reliability: Cohen's kappa = 0.72; 95% confidence interval (CI) (0.53–0.91)].

Among the 21 articles, the data provided in three studies (Boyle et al., 2007; Ebbels et al., 2007; Dickson et al., 2009) were unavailable and therefore were also excluded. One study (Boyle et al., 2009) involved data re-analysis and therefore was excluded. In addition, by reviewing Law et al.'s meta-analysis (Law et al., 2004), one study (Girolametto et al., 1996) was added to the considered publications.

### Quality assessment

The methodological quality of the included trials was assessed based on the modified Jadad scale (Table 1). Among the included studies, five studies (Almost and Rosenbaum, 1998; Ebbels et al., 2012; Wake et al., 2013; Buschmann et al., 2015; Hampton et al., 2017) had a low risk of bias, 11 studies (Girolametto et al., 1996, 1997; Boyle et al., 2007, 2009; Dickson et al., 2009; Smith-Lock et al., 2013; Ebbels, 2014; Buschmann et al., 2015; Cleave et al., 2015; Wu et al., 2017; Xu et al., 2018) had a moderate risk of bias, and two (Ebbels et al., 2012; Wake et al., 2013) had a high risk of bias. To avoid possible negative impact upon the outcome of this study, we excluded the studies at a high risk of bias from further analysis. The inter-rater reliability of publication quality assessment was fair to very good [Cohen's kappa = 0.63; 95% CI (0.37–0.89)].

**Table 1:** Quality assessment of the publications included in this study using the modified Jadad scale.

	**Randomization**			**Allocation concealment**				**Blinding**			**Withdrawals/dropouts**	**Total score**
**Author(s), year**	**Adequate**	**Unclear**	**Inadequate**	**Adequate**	**Unclear**	**Inadequate**	**None**	**Adequate**	**Unclear**	**Inadequate or unused**	**(Yes/no)**	
Gibbard (1994)		1			1			2			1	5
Girolametto et al. (1996)		1			1			2			0	4
Girolametto et al. (1997)		1			1				1		1	4
Almost and Rosenbaum (1998)	2			2				2			1	7
Robertson and Weismer (1999)		1			1				1		1	4
Glogowska et al. (2000)		1		2						0	1	4
Denne et al. (2005)		1		2					1		1	5
Boyle et al. (2007)		‘			1			1			1	4
Gallagher and Chiat (2009)		1					0	2			1	3
Ebbels et al. (2012)	2				1			2			1	6
Smith-Lock et al. (2013)			0		1	0		2			1	4
Wake et al. (2013)	2			2				2			1	7
Buschmann et al. (2015)	2			2				2			1	7
Roberts and Kaiser (2015)	2			2						0	1	5
Lee and Pring (2016)		1			1			2		1		5
Dawes et al. (2019)		1			1			2			0	4
Hampton et al. (2017)	2			2						1	1	6
Kruythoff-Broekman et al. (2019)		1					0			0	1	2

### Descriptive characteristics of the included studies

Among the 15 studies that were suitable for further analysis, the youngest clients were aged 22–33 months (Girolametto et al., 1997; Cleave et al., 2015), whereas the oldest were aged 9–15 years (Ebbels et al., 2012) (the reason for this inclusion was that this study contained detailed raw data based on which the children satisfying the age criterion in this study could be selected for analysis). The shortest intervention was 8 weeks (Denne et al., 2005; Ebbels et al., 2012; Smith-Lock et al., 2013; Dawes et al., 2019) while the longest was 10 months (Wake et al., 2013). Four studies involved parent-guided intervention (Gibbard, 1994; Girolametto et al., 1996, 1997; Hampton et al., 2017), whereas the remaining involved nonparent-guided intervention. The smallest sample size was found in Ebbels et al.'s study (Ebbels et al., 2012).

After careful examination, six studies could be analyzed at the level of overall expressive language, three studies at the level of the mean length of utterances in a language sample, three studies at the level of the number of utterances in a language sample, three studies at the level of parent reports of expressive phrase complexity, two at the level of expressive grammar ability, three at the level of the proportion of consonants correct in conversation or storytelling, four at the level of the overall vocabulary development, four at the level of different words used in a language sample, two at the level of parent reports of vocabulary, and six at the level of overall receptive language development. Three studies could be analyzed for the effect of parent-guided intervention and three for the effect of nonparent-guided intervention. In addition, two studies could be used to show the long-term effect of intervention on overall expressive language development and four for the long-term effect of intervention on overall receptive language development.

The detailed descriptive characteristics of the included studies are provided in Supplementary Table 1.

### Immediate effect of intervention on language development

#### Effect of intervention on expressive language development

**Figure 1:**
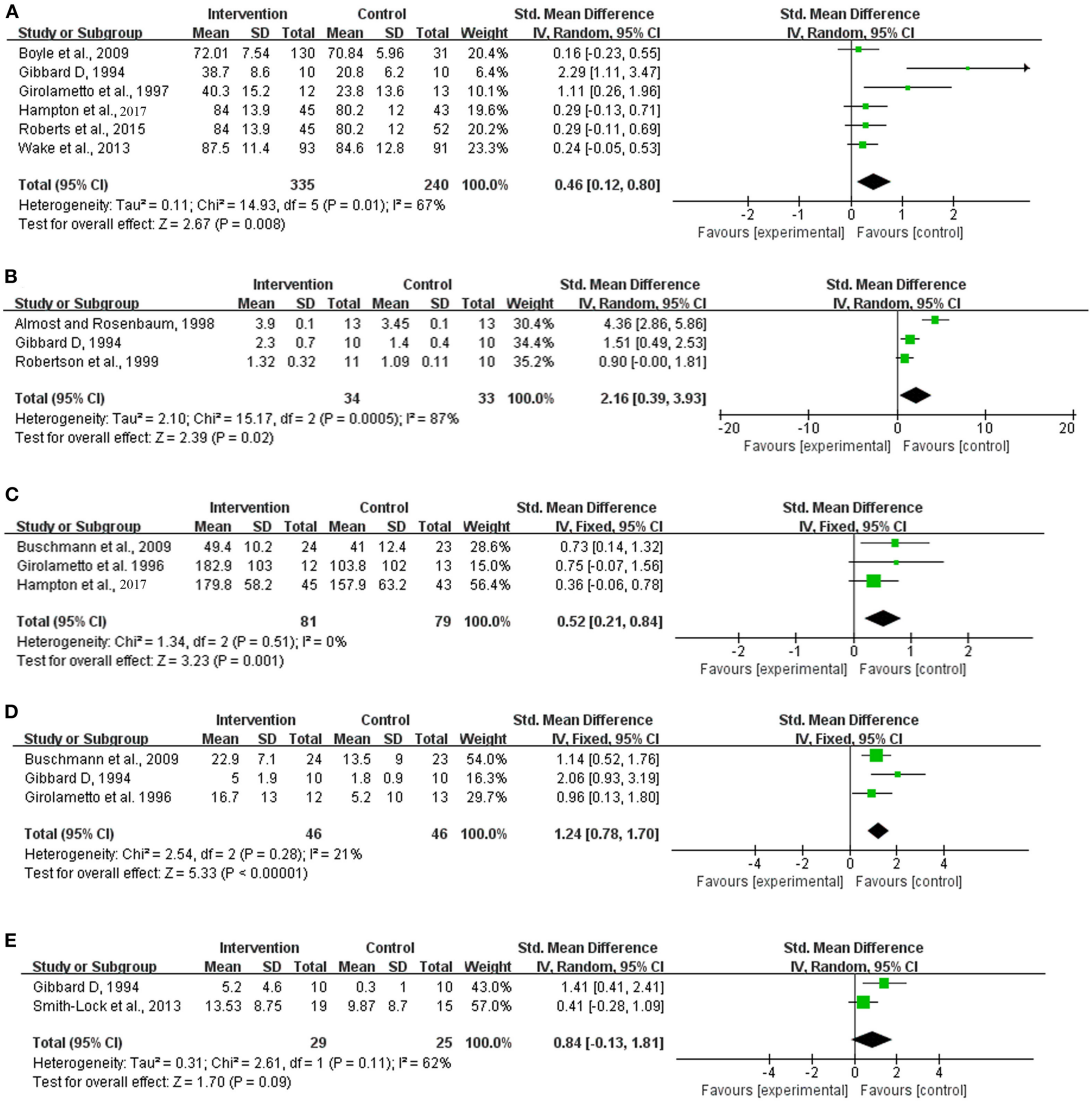
Meta-analysis outcomes of the intervention for expressive language development. **(A)** Overall expressive language; **(B)** Mean length of utterances in a language sample; **(C)** Number of utterances in a language sample; **(D)** Parent reports of expressive phrase complexity; **(E)** Expressive grammar ability. Control, no or delayed treatment.

(1) Overall expressive language development. At the overall expressive language level, six trials qualified for summary statistics. The results of the six independent trials were not homogeneous (Chi^2^ = 14.93, df = 5 [*p* = 0.01]; *I*^2^ = 67%) and therefore required the use of a random effects model. The meta-analysis suggested a significant difference in overall expressive language development between the intervention and control groups, with the latter group receiving no or delayed intervention (SMD = 0.46, 95% CI = 0.12–0.80 [*p* = 0.008]) (Figure 1A).

(2) Expressive syntax development. The literature searches showed that meta-analyses could be performed only at the levels of the mean length of utterances (MLU) in a language sample, number of utterances (NU) in a language sample, parent reports of expressive phrase complexity (PREPC) and expressive grammar ability in this regard.

The results of the independent trials at the levels of MLU (Chi^2^ = 15.17, df = 2 [*p* = 0.0005]; *I*^2^ = 87%) and expressive grammar ability (Chi^2^ = 2.61, df = 1 [*p* = 0.11]; *I*^2^ = 62%) showed strong heterogeneity, thus requiring a random effects model. In contrast, the results at the levels of NU (Chi^2^ = 1.34, df = 2 [*p* = 0.51]; *I*^2^ = 0%) and PREPC (Chi^2^ = 2.54, df = 2 [*p* = 0.28]; *I*^2^ = 21%) showed satisfactory homogeneity, indicating the use of a fixed effects model. The meta-analyses indicated significant differences in the MLU (SMD = 2.16, 95% = 0.39–3.93 [*p* = 0.02]; Figure 1B), NU (SMD = 0.52, 95% CI = 0.21–0.84 [*p* = 0.001]; Figure 1C) and PREPC (SMD = 1.24, 95% CI = 0.78–1.70 [*p* < 0.001]; Figure 1D) between the two groups, but no significant difference in expressive grammar ability was observed (SMD = 0.84, 95% CI = −0.13–1.81 [*p* = 0.09]; Figure 1E).

(3) Expressive phonological development. The proportion of consonants that were correct in conversation or storytelling was the only expressive phonological development index that could be introduced to a meta-analysis, since other indices, such as the average phonological deviations and number of phonological errors, were detailed in only one article among all those considered in this study.

**Figure 2:**
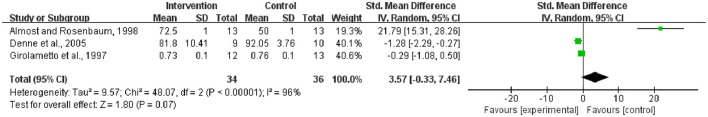
Meta-analysis outcome of the intervention effect on the proportion of consonants correct in conversation or storytelling. Control, no or delayed treatment.

The results of the independent trials at the levels of proportion of consonants correct in conversation or storytelling showed strong heterogeneity among the included trials (Chi^2^ = 48.07, df = 2 [*p* < 0.00001]; *I*^2^ = 96%; Figure 2). Therefore, a random effects model was determined for this summary statistic. No significant difference was observed in this index between the two groups (SMD = 3.57, 95% CI = −0.33–7.46 [*p* = 0.07]).

(4) Expressive vocabulary development. Summary statistics could be obtained at the levels of overall vocabulary development, different words used in a language sample and parent reports of vocabulary.

**Figure 3:**
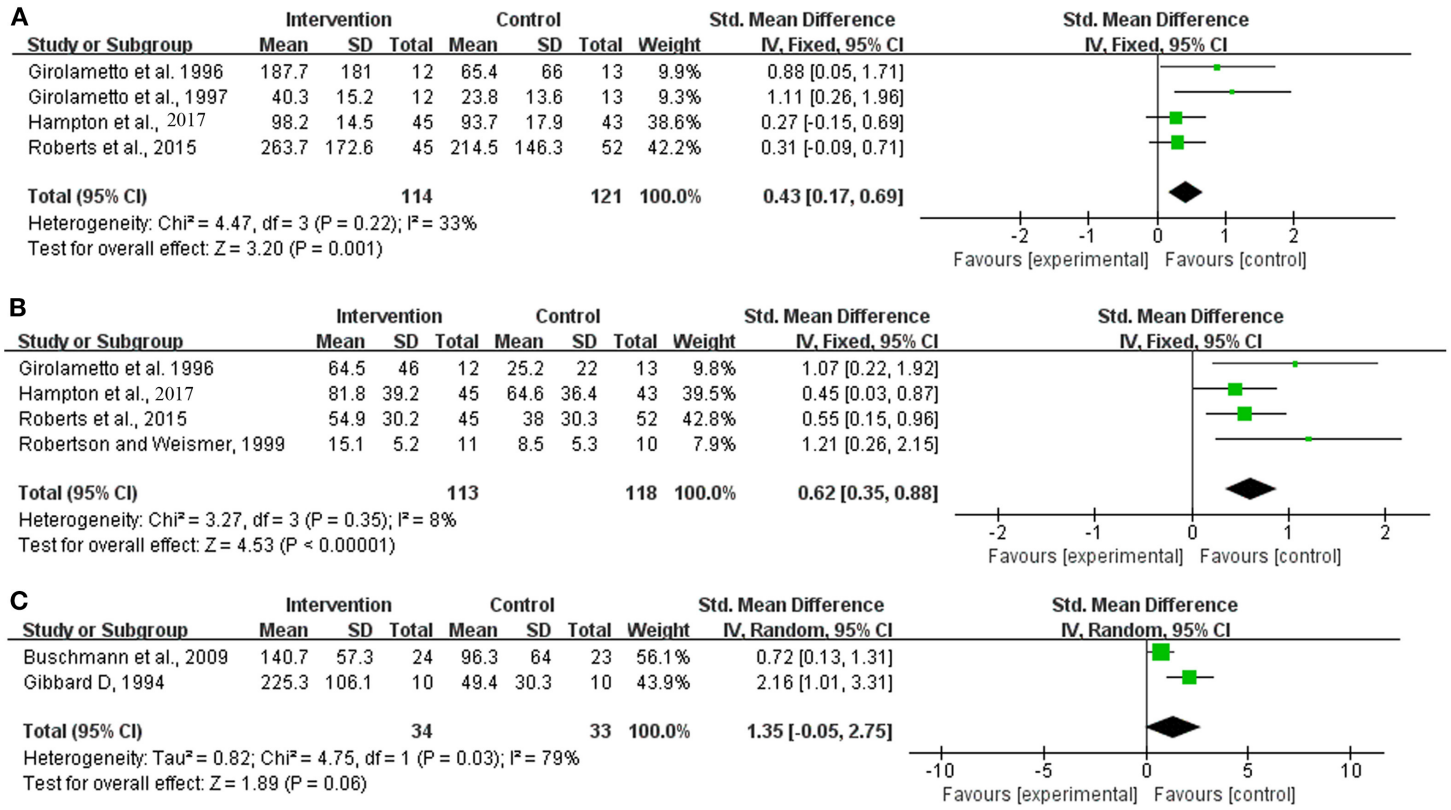
Meta-analysis outcome of the intervention effect on expressive vocabulary development. **(A)** Overall vocabulary development; **(B)** Different words used in a language sample; **(C)** Parent reports of vocabulary. Control, no or delayed treatment.

The heterogeneity test showed that fixed effects models were required for the analyses of overall vocabulary development (Chi^2^ = 4.47, df = 3 [*p* = 0.22]; *I*^2^ = 33%) and different words used in a language sample (Chi^2^ = 3.27, df = 3 [*p* = 0.35]; *I*^2^ = 8%), whereas a random effects model was needed for the analysis of parent reports of vocabulary (Chi^2^ = 4.75, df = 1 [*p* = 0.03]; *I*^2^ = 8%) (Figure 3).

Significant differences were observed in the overall vocabulary development and different words used in a language sample, both in favor of the intervention group (SMD = 0.43, 95% CI = 0.17–0.69 [*p* = 0.001] and SMD = 0.62, 95% CI = 0.35–0.88 [*p* < 0.001]). No significant between-group difference was observed in the parent reports of vocabulary (SMD = 1.35, 95% CI = −0.05–2.75 [*p* = 0.06]).

#### Receptive language development

Among various receptive language levels, overall receptive language development was the only index that could be analyzed based on the included trials.

**Figure 4:**
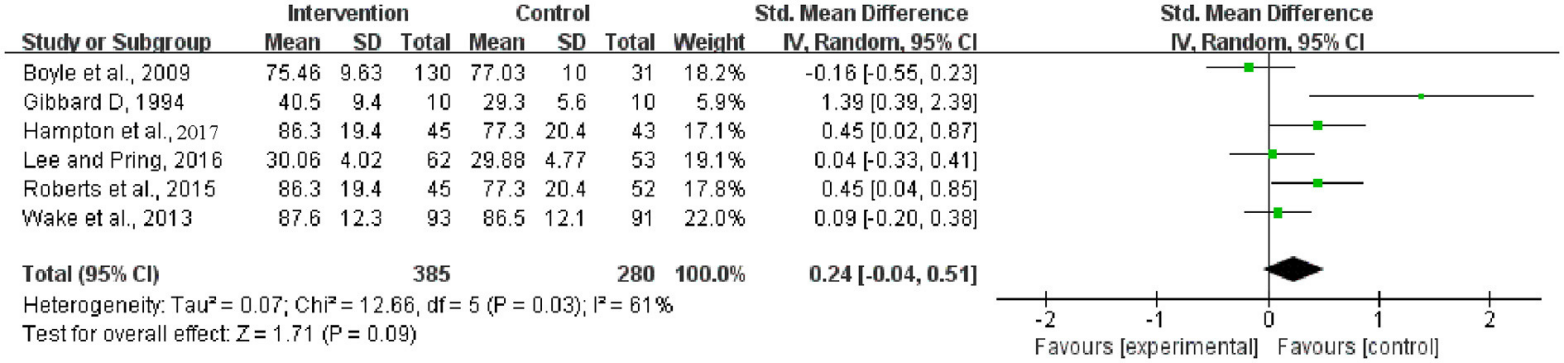
Meta-analysis outcome of the intervention effect on overall receptive language development. Control, no or delayed intervention.

According to the heterogeneity test, a random effects model was required (Chi^2^ = 12.66, df = 5 [*p* = 0.03]; *I*^2^ = 61%; Figure 4). No significant difference in overall receptive language development was observed between the groups (SMD = 0.24, 95% CI = −0.04–0.51 [*p* = 0.09]).

#### Intervention effect comparisons: Parent vs. therapist/assistant/clinician (non-parent)

The intervention effects of the parent- and non-parent-directed methods were compared. Notably, although some interventions adopted in the included trials were parent guided, all were performed after parent training. In addition, considering that a rather small number of trials were included in this study, the stratification analyses here were performed only at the levels of overall expressive language development and overall receptive language development.

**Figure 5:**
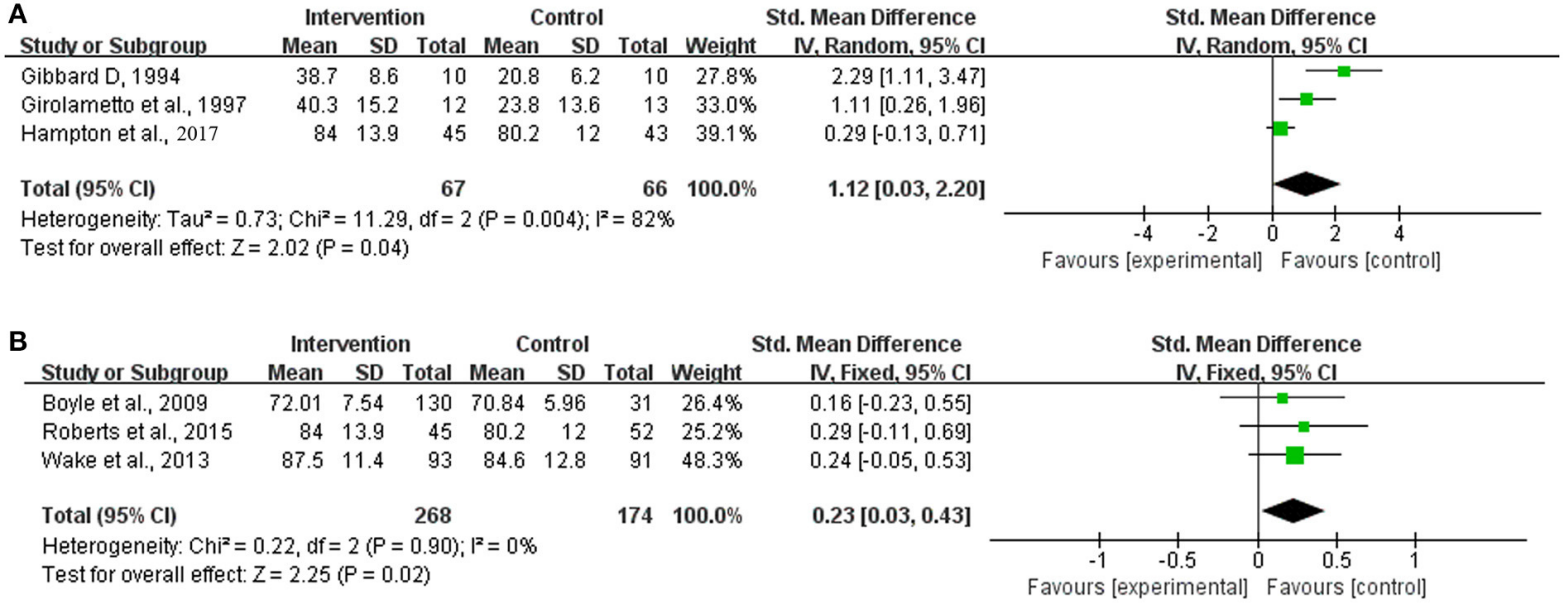
Meta-analysis outcomes of the effect of the parent-guided intervention and non-parent-guided intervention on overall expressive language development. **(A)** Parent-guided intervention; **(B)** Non-parent-guided intervention. Control, no or delayed intervention.

The results of the three independent trials for the analysis of the parent-guided intervention for overall expressive language development were highly heterogeneous (Chi^2^ = 11.29, df = 2 [*p* = 0.004]; *I*^2^ = 82%) and therefore indicated the need for a random effects model. The results of the three independent trials for the analysis of the non-parent-guided intervention for overall expressive language development were not heterogeneous (Chi^2^ = 0.22, df = 2 [*p* = 0.90]; *I*^2^ = 0%) and therefore indicated the need for a fixed effects model. The results showed that both the parent-guided and non-parent-guided interventions achieved significant improvements in overall expressive language development compared with the development observed for the corresponding control groups (SMD = 1.12, 95% CI = 0.03–2.20 [*p* = 0.04] and SMD = 0.23, 95% CI = 0.03–0.43 [*p* = 0.02], respectively) (Figure 5).

**Figure 6:**
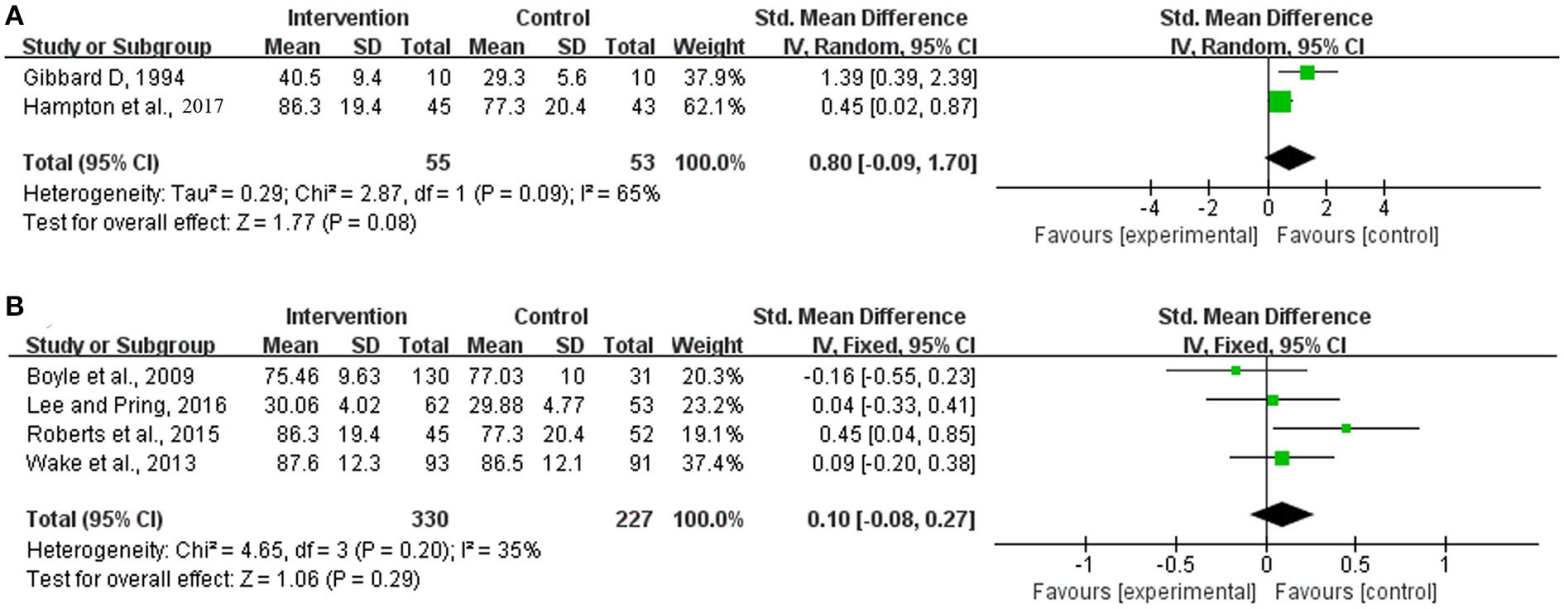
Meta-analysis outcomes of the effect of the parent-guided intervention and non-parent-guided intervention on overall receptive language development. **(A)** Parent-guided intervention; **(B)** Non-parent-guided intervention. Control, no or delayed intervention.

The results of the independent trials for the analysis of the parent-guided intervention for overall receptive language development indicated strong heterogeneity (Chi^2^ = 2.87, df = 1 [*p* = 0.09]; *I*^2^ = 65%), whereas those for the analysis of the non-parent-guided intervention showed satisfactory homogeneity (Chi^2^ = 4.65, df = 3 [*p* = 0.20]; *I*^2^ = 35%). The summary statistics showed that neither intervention method achieved significant improvements in overall respective language development compared with the development observed for the corresponding control groups (SMD = 0.80, 95% CI = −0.09–1.70 [*p* = 0.08] and SMD = 0.10, 95% CI = −0.08–0.27 [*p* = 0.29], respectively) (Figure 6).

### Long-term effect of intervention

Three of the trials in this study also endeavored to investigate the long-term effect of the intervention by follow-up, and the time intervals from the completion of the intervention to the last follow-up ranged from 3.5 months to 1.5 years. Based on the included studies, only overall expressive and receptive language development could be analyzed.

**Figure 7:**
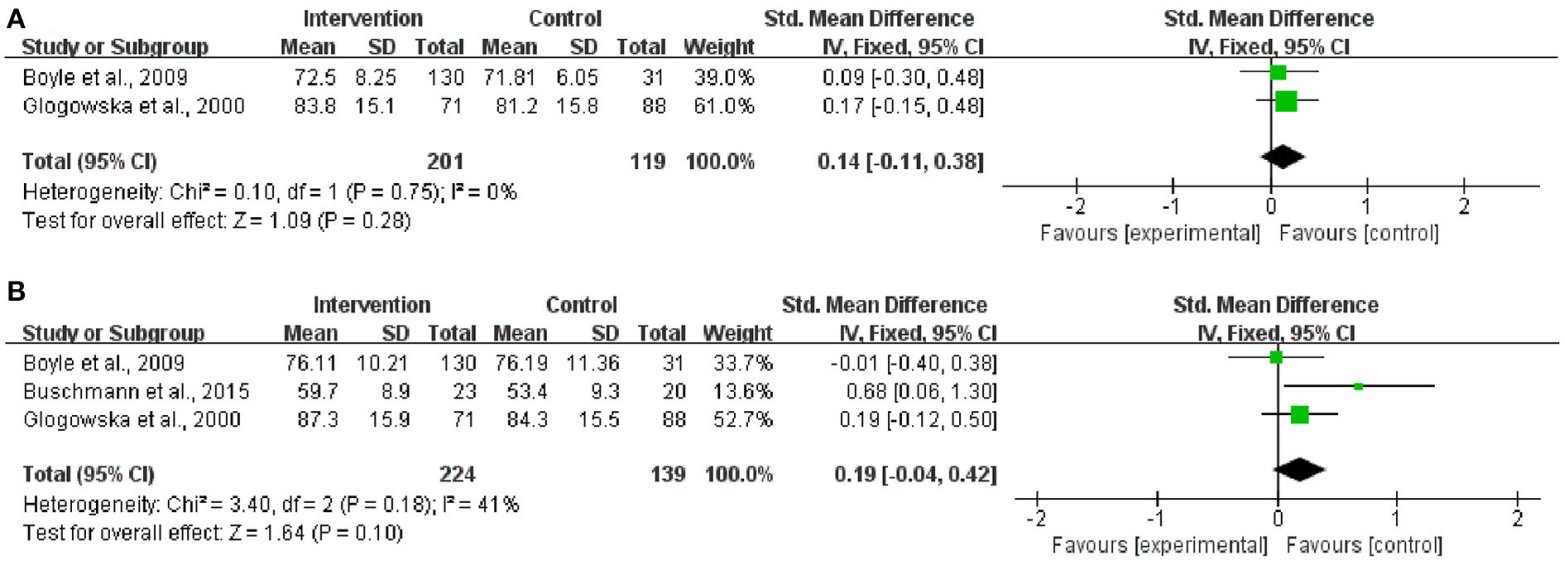
Meta-analysis outcomes of the long-term effect of the intervention on overall expressive and receptive language development. **(A)** Overall expressive language development; **(B)** Overall receptive language development. Control, no or delayed intervention.

According to the heterogeneity test, both the included trials for the analysis of overall expressive language development and those for the analysis of overall receptive language development showed satisfactory homogeneity ([Chi^2^ = 0.10, df = 1 [*p* = 0.75]; *I*^2^ = 0%] and [Chi^2^ = 3.40, df = 2 [*p* = 0.18]; *I*^2^ = 41%], respectively) (Figure 7). Compared to the corresponding control group, the intervention group did not show a significant difference in either of the considered indices (expressive language development: SMD = 0.14, 95% CI = −0.11–0.38 [*p* = 0.28]; receptive language development: SMD = 0.19, 95% CI = −0.04–0.42 [*p* = 0.10]).

### Publication bias

**Figure 8:**
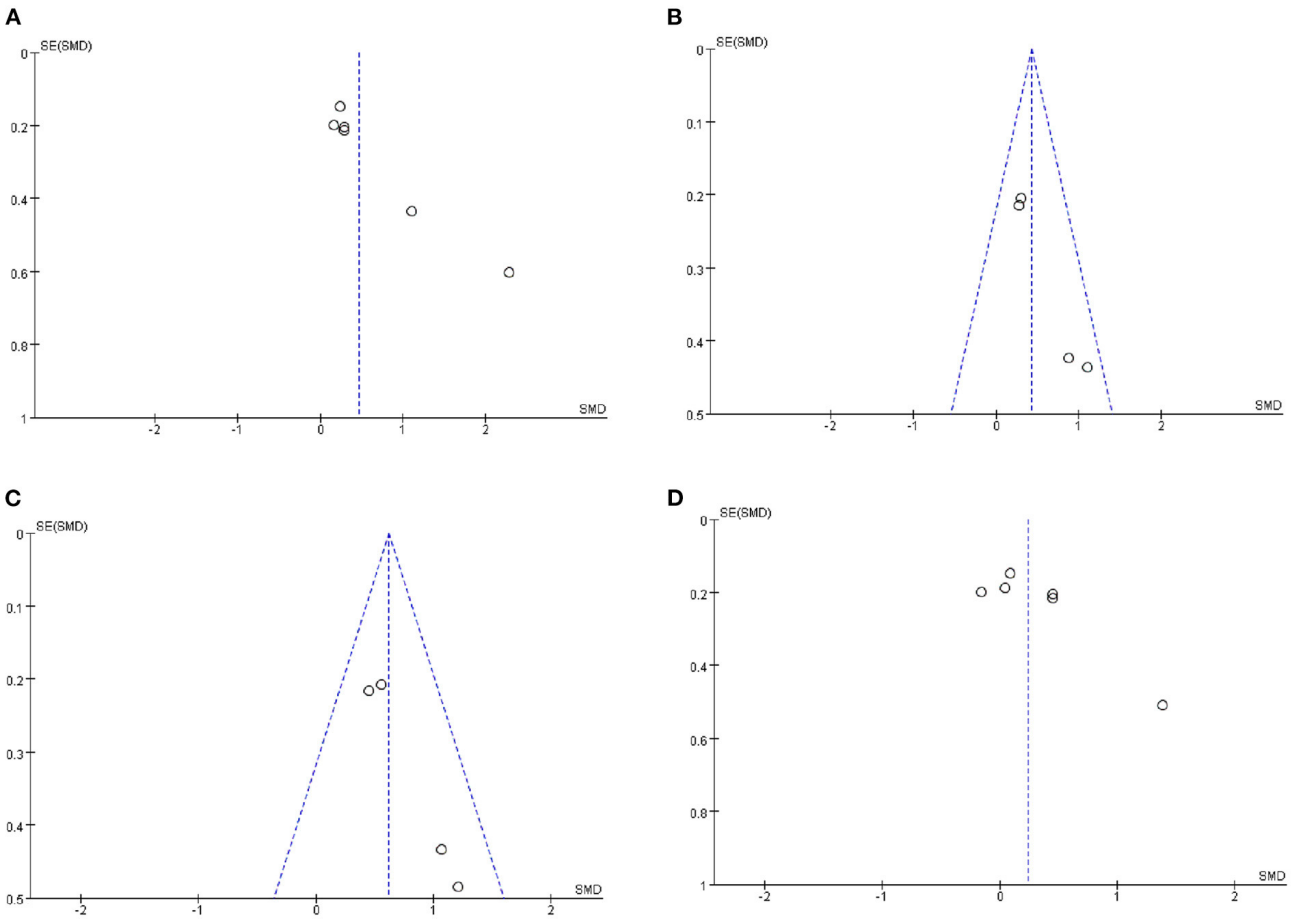
Funnel plot of the included trials for the analyses at different language levels. **(A)** Overall expressive language development. **(B)** Overall vocabulary development. **(C)** Different words used in language sample. **(D)** Overall receptive language development.

Publication bias was analyzed based on funnel plots. As shown in Figure 8, at the overall expressive language development level, the included trials were in satisfactory symmetry; at the levels of overall expressive language development (Figure 8A), overall vocabulary development (Figure 8B) and different words used in a language sample (Figure 8C), a quite satisfactory symmetry could also be observed, except for sample size. Due to the small number of the included studies, publication bias analyses were not performed at the remaining levels.

## Discussion

DLD has a high incidence among preschool children. It can be manifested across a wide range of linguistic domains, such as phonology, lexical semantics, morphosyntax, pragmatics (although controversial) and suprasegments, showing peculiarities according to individuals. Therefore, a better understanding of the linguistic properties of DLD is of both clinical and theoretical significance because this knowledge will provide useful information for clinical therapists about what linguistic domains should be targeted as well as for scholars to divide the whole linguistic region into smaller plots where in-depth research can be performed. In this study, we investigated the effect of language intervention at various linguistic levels in children with DLD based on the meta-analyses of 15 RCTs. We also analyzed the long-term effects of interventions for DLD based on summary statistics. Although a few systematic reviews focused on a similar topic (Ney et al., 1987; Law et al., 1998, 2003; 2004), all focused on the immediate effect of the interventions on various linguistic levels in children with DLD and, to date, those focusing on the long-term effect after some time interval from the completion of the intervention have not been found in the literature.

Although a large number of related publications were obtained after initial screening, a total of 15 trials were ultimately included. This number was small and did not show an advantage compared with that included in the meta-analysis conducted by Law et al. (2004). The inclusion of such a small number of trials could be partially attributed to the strict inclusion/exclusion criteria. In this study, we considered only randomized case-control trials and excluded those involving self-controlled trials, such as those by Smith-Lock et al. (2015) and by Cole and Dale (1986). This exclusion criterion was based on the following consideration: Language competence and performance in DLD children can also develop in a naturalistic context with age, and a certain proportion of these children may even catch up with reference norms. Therefore, the inclusion of these self-controlled trials could not indicate how many benefits should be primarily credited to language intervention alone. Furthermore, we excluded all trials involving bilinguals with DLD, such as the study conducted by Shelton et al. (1978), as the “silent period” may serve as an influential factor in children's performance and competence at the early stage of language development. In addition, we set “an intervention period of no shorter than 8 weeks” as an inclusion criterion, which was based on the analytical result described in Law et al.'s study (2004). As a result, trials that involved an intervention period shorter than 8 weeks and were included in Law et al.'s meta-analysis were, however, excluded from consideration in the present study.

The summary statistics of this study showed that in terms of the immediate effect (via a language test performed once the intervention was completed), language intervention could improve DLD children's language competence and performance at most levels of expressive language, such as overall expressive language development [standard mean differences (SMD) = 0.46, 95% confidence interval (CI) = 0.12–0.80], mean length of utterances in a language sample (SMD = 2.16, 95% = 0.39–3.93), number of utterances in a language sample (SMD = 0.52, 95% CI = 0.21–0.84), parent reports of expressive phrase complexity (SMD = 1.24, 95% CI = 0.78–1.70), overall expressive vocabulary development (SMD = 0.43, 95% CI = 0.17–0.69) and different words used in a language sample (SMD = 0.62, 95% CI = 0.35–0.88). However, such an improvement was not observed at the levels of expressive grammar ability and the proportion of consonants correct in conversation and storytelling, parent reports of vocabulary and overall receptive language development. Our results almost completely contradicted those reported by Law et al. (2004). In their study, expressive phonology, different words in the language sample and parent reports of vocabulary were the linguistic levels where language intervention came into play. The contradiction between our results and theirs may be explained as follows. In Law et al.'s study, they did not set an intervention time span-related exclusion criterion; that is, they included both trials with an intervention time over 8 weeks and those with an intervention time shorter than 8 weeks. In their intervention duration-based stratification analysis, they reported that an intervention duration over 8 weeks seemed to be more effective than an intervention of <8 weeks. Therefore, the integration of trials with a relatively short intervention duration might partially conceal the due effect of language intervention for DLD. Furthermore, the inclusion of trials involving a self-control method in their study could not exclude the possibility that the children's development mitigated the severity of DLD with age, which further obscured the effectiveness of language intervention.

Parents play a crucial role in children's language development (Smith-Lock et al., 2013), and thus, the participation of the parents of children with DLD has been assumed to greatly benefit children. In this study, we also adopted a stratification strategy to separately investigate the effect of parent-directed intervention (although these parents had been advised on how to facilitate their children's language difficulties before recruitment) and non-parent-directed intervention. The results showed that both parent-directed and non-parent-directed interventions improved the children's performance at the levels of overall expressive and receptive language development. In Law et al. (2004) study, the clinician-based and parent-based interventions did not differ significantly in the measures of overall expressive syntax development (SMD = −0.04, 95% CI = −0.56–0.48), the measures of overall expressive vocabulary development (SMD = 0.20, 95% CI = −0.40–0.79) or the measures of overall receptive syntax development (SMD = −0.11, 95% CI = −0.87–0.65). Our results were basically consistent with those reported by Law et al.

In addition, we investigated the long-term effect of language intervention on language development in DLD children. After careful examination, three trials were eligible for this analysis. The time span from the completion of the intervention to the last follow-up in these studies ranged from 3.5 months to 1.5 years. Our results did not show significant differences in expressive and receptive language development between the intervention group and the corresponding control group (SMD = 0.14, 95% CI = −0.11–0.38 and SMD = 0.19, 95% CI = −0.04–0.42, respectively). Our literature searches did not reveal any systematic reviews with this aim. These negative outcomes of the meta-analyses may pose great challenges for both language therapists and linguists. Do these outcomes indicate that we still cannot find the right pathway to provide intervention for DLD because what we have already known about language is far from sufficient? Do the findings indicate that the most effective method remains to be determined, considering that the involved trials adopted a variety of intervention methods? The answers to these questions need to be explored in the future. What should also be noteworthy was that in this meta-analysis, we failed to investigate the intervention effect in linguistic domains at the receptive level, and overall receptive language was the only analyzable index in this respect. Although this imbalance between research on expressive DLD and on receptive DLD might be partially attributed to the small number of included publications in this study, it suggested on its own the lack of good-quality research on interventions into receptive DLD in the literature (Law et al., 2004; Ebbels, 2014). Therefore, more research at this level remains to be conducted in the future.

However, this systematic review did have limitations. First, the sample size in this meta-analysis was small, which may cause bias in the results of this study. This limitation, as well as the lack of one-to-one pre-post-treatment values in the reported literature, precluded us from further performing stratification analyses to calculate the possible difference in post-intervention gains between sexes and among different age groups. In the future, more randomized case-control studies concerning the intervention effect on DLD should be conducted. Second, the reported studies themselves may have impact upon the outcomes of this meta-analysis. Although part of the funnel plots (Figure 8) showed that the included trials were in satisfactory symmetry, they were different in sample size. Even though we implemented rather strict inclusion/exclusion criteria to select the trials, the included trials still differed greatly in their methods. For instance, the six publications included in the analysis of overall expressive language development differed greatly in sample size, means of stimulation (focused vs. naturalistic), implementation types (parent directed vs. non-parent directed), intervention durations (ranging from 3 to 10 months), and assessment approaches (such as the Preschool Language Scale–Fourth edition (PLS-4) (Hampton et al., 2017), the Clinical Evaluation of Language Fundamentals (CELF)-3^UK^-Expressive (Boyle et al., 2007), MacArthur Communicative Development Inventories (Girolametto et al., 1996), Reynell Developmental Language Scales and Clinical Evaluation of Language Fundamentals—Preschool, 2nd Edition; Gibbard, 1994; Supplementary Table 1). Third, apart from the small sample size included in this study as well as the heterogeneity of the test methods reported in the published articles, other factors might also bring bias to the interpretation of the results of this study. DLD, although frequent in children, is often challenging to detect, particularly in children. Therefore, the accuracy of the data reported in previous studies might not be fully guaranteed. Additionally, DLD itself exhibits peculiarities in manifestations as well as in response to intervention according to individuals (Kapa et al., 2020). Therefore, the findings of this meta-analysis should be cautiously treated. Fourth, due to a small number of included studies at each level of analysis, as well as because we removed all studies at a high risk of bias to avoid possible negative impact on our final outcomes, we did not perform sensitivity analysis, which constituted another limitation of our study. In addition, the known limitation of meta-analysis is that positive results tend to be more frequently reported than negative results, which may also have led to bias in the current study.

## Concluding remarks

In this study, we investigated the effect of language intervention alone on DLD at different linguistic levels, and for the first time, we obtained summary statistics at the level of the long-term intervention effect. Our results showed that language intervention has a positive immediate effect at most levels of expressive language. However, in regard to long-term effects, this improvement effect is greatly compromised. Despite the rather disappointing results of this meta-analysis, some valuable and promising research can be conducted in the future.

First, the unsatisfactory long-term effect of intervention on DLD may be due to the poor understanding of this condition. To date, the etiology of DLD remains mysterious, which may suggest that the best pathway to intervene in this condition has not been found yet. Therefore, much work remains to be done to investigate its pathogenesis. Scholars normally conduct explorations of environmental and genetic factors for a disease. Similarly, the causes of DLD may lie on the continuum between environmental factors (impoverished speech input) and genetic factors. However, the pathogenesis of DLD remains understudied currently. In terms of environmental factors, great controversy remains (Wang et al., 2018). Future studies with a larger sample size can be conducted to further explore the independent risk factors for DLD. In terms of genetic factors, we have come to describe the nature of this condition as “being primary.” Although some scholars use “primary” and “specific” to describe the genetic nature of DLD, school-age children with DLD seem to present with lower academic performance than norm-referenced children (Bishop and Clarkson, 2003). If DLD is not “primary” in nature, bioinformatics studies or genome-wide association analysis may be promising to explore the genetic spectrum of these children on the condition that environmental risk factors for DLD are well-controlled. Another reason to explain the unsatisfactory long-term effects of interventions for DLD is that scholars and researchers pay relatively little attention to this effect compared to the immediate effect. To solve this problem, efforts are needed from language therapists as well as linguists.

## Data availability statement

The original contributions presented in the study are included in the article/Supplementary material, further inquiries can be directed to the corresponding author.

## Author contributions

SF devised the study plan, led the writing, and performed analysis. XS and YW were responsible for data collection and validation. BM supervised the entire process and reviewed the draft. All authors contributed to the article and approved the submitted version.

## Conflict of interest

The authors declare that the research was conducted in the absence of any commercial or financial relationships that could be construed as a potential conflict of interest.

## Publisher's note

All claims expressed in this article are solely those of the authors and do not necessarily represent those of their affiliated organizations, or those of the publisher, the editors and the reviewers. Any product that may be evaluated in this article, or claim that may be made by its manufacturer, is not guaranteed or endorsed by the publisher.

## References

[B1] Allen J. Marshall C. R. (2011). Parent–child interaction therapy in school-aged children with specific language impairment. Int. J. Lang. Commun. Disord. 46, 397–410. 10.3109/13682822.2010.517600 21771216

[B2] Almost D. Rosenbaum P. (1998). Effectiveness of speech intervention for phonological disorders: a randomized controlled trial. Dev. Med. Child Neurol. 40, 319–325. 10.1111/j.1469-8749.1998.tb15383.x 9630259

[B3] Bioshop D. V. M. Snowling M. J. Thompson P. A. Greenhalgh T. and the CATALISE-2 consortium. (2017). Phase 2 of CATALISE: a multinational and multidisciplinary Delphi consensus study of problems with language development: terminology. J. Psychol. Psychiatry. 58, 1068–1080. 10.7287/peerj.preprints.2484v2 28369935 PMC5638113

[B4] Bishop D. V. Clarkson B. (2003). Written language as a window into residual lan¬guage deficits: a study of children with persistent and residual speech and language impairments. Cortex 39, 215–237. 10.1016/S0010-9452(08)70106-0 12784886

[B5] Boyle J. McCartney E. Forbes J. O'Hare A. (2007). A randomised controlled trial and economic evaluation of direct versus indirect and individual versus group modes of speech and language therapy for children with primary language impairment. Health Technol. Assess. 11, iii–iv, xi–xii, 1–139. 10.3310/hta11250 17610807

[B6] Boyle J. M. McCartney E. O'Hare A. Forbes J. (2009). Direct versus indirect and individual versus group modes of language therapy for children with primary language impairment: principal outcomes from a randomized controlled trial and economic evaluation. Int. J. Lang. Commun. Disord. 44, 826–846. 10.1080/13682820802371848 19107656

[B7] Buschmann A. Multhauf B. Hasselhorn M. Pietz J. (2015). Long-term effects of a parent-based language intervention on kanguage outcomes and working memory for late-talking toddlers. J. Early Interv. 37, 175–189. 10.1177/1053815115609384

[B8] Cleave P. L. Becker S. D. Curran M. K. Van Horne A. J. Fey M. E. (2015). The efficacy of recasts in language intervention: a systematic review and meta-analysis. Am. J. Speech Lang. Pathol. 24, 237–255. 10.1044/2015_AJSLP-14-0105 25654306 PMC4450887

[B9] Cole K. N. Dale P. S. (1986). Direct language instruction and interactive language instruction with language delayed preschool children: a comparison study. J. Speech Hear. Res. 29, 206–217. 10.1044/jshr.2902.206 3724113

[B10] Dawes E. Leitão S. Claessen M. Kane R. (2019). A randomized controlled trial of an oral inferential comprehension intervention for young children with developmental language disorder. Child Lang. Teach. Ther. 35, 39–54. 10.1177/0265659018815736

[B11] Denne M. Langdown N. Pring T. Roy P. (2005). Treating children with expressive phonological disorders: does phonological awareness therapy work in the clinic? Int. J. Lang. Comm. Dis. 40, 493–504. 10.1080/13682820500142582 16195202

[B12] Dickson K. Marshall M. Boyle J. McCartney E. O'Hare A. Forbes J. (2009). Cost analysis of direct versus indirect and individual versus group modes of manual-based speech-and-language therapy for primary school-age children with primary language impairment. Int. J. Lang. Commun. Disord. 44, 369–381. 10.1080/13682820802137041 18821106

[B13] Ebbels S. (2014). Effectiveness of intervention for grammar in school-aged children with primary language impairments: a review of the evidence. Child Lang. Teach. Ther. 30, 7–40. 10.1177/0265659013512321

[B14] Ebbels S. H. Nicoll H. Clark B. Eachus B. Gallagher A. L. Horniman K. . (2012). Effectiveness of semantic therapy for word-finding difficulties in pupils with persistent language impairments: a randomized control trial. Int. J. Lang. Commun. Disord. 47, 35–51. 10.1111/j.1460-6984.2011.00073.x 22268900

[B15] Ebbels S. H. van der Lely H. K. Dockrell J. E. (2007). Intervention for verb argument structure in children with persistent SLI: a randomized control trial. J. Speech Lang. Hear. Res. 50, 1330–1349. 10.1044/1092-4388(2007/093) 17905915

[B16] Fan S. F. Zhang Y. Qin J. B. Song X. Wang M. Ma J. (2021). Family environmental risk factors for developmental speech delay in children in Northern China. Sci. Rep. 11, 3924. 10.1038/s41598-021-83554-w 33594136 PMC7887192

[B17] Frizelle P. Tolonen A. K. Tulip J. Murphy C. A. Saldana D. McKean C. (2021a). The influence of quantitative intervention dosage on oral language outcomes for children with developmental language disorder: a systematic review and narrative synthesis. Lang. Speech Hear. Serv. Sch. 52, 738–754. 10.1044/2020_LSHSS-20-00058 33465314

[B18] Frizelle P. Tolonen A. K. Tulip J. Murphy C. A. Saldana D. McKean C. (2021b). The impact of intervention dose form on oral language outcomes for children with developmental language disorder. J. Speech Lang. Hear. Res. 64, 3253–3288. 10.1044/2021_JSLHR-20-00734 34213951

[B19] Gallagher A. L. Chiat S. (2009). Evaluation of speech and language therapy interventions for pre-school children with specific language impairment: a comparison of outcomes following specialist intensive, nursery-based and no intervention. Int. J. Lang. Commun. Dis. 44, 616–638. 10.1080/13682820802276658 19424890

[B20] Ghaffari M. Rakhshanderou S. Ramezankhani A. Noroozi M. Armoon B. (2018). Oral health education and promotion programmes: meta-analysis of 17-year intervention. Int. J. Dent. Hyg. 16, 59–67. 10.1111/idh.12304 28836347

[B21] Gibbard D. (1994). Parental-based intervention with pre-school language-delayed children. Eur. J. Disord. Commun. 29, 131–150. 10.3109/13682829409041488 7865920

[B22] Girolametto L. Pearce P. S. Weitzman E. (1996). Interactive focused stimulation for toddlers with expressive vocabulary delays. J. Speech Hear. Res. 39, 1274–1283. 10.1044/jshr.3906.1274 8959612

[B23] Girolametto L. Pearce P. S. Weitzman E. (1997). Effects of lexical intervention on the phonology of late talkers. J. Speech Lang. Hear. Res. 40, 338–348. 10.1044/jslhr.4002.338 9130202

[B24] Glogowska M. Roulstone S. Enderby P. Peter T. J. (2000). Randomised controlled trial of community based speech and language therapy in preschool children. Br. Med. J. 321, 923–926. 10.1136/bmj.321.7266.923 11030677 PMC27499

[B25] Hampton L. H. Kaiser A. P. Roberts M. Y. (2017). One-year language outcomes in toddlers with language delays: an RCT follow-up. Pediatrics 140, e20163646. 10.1542/peds.2016-3646 29054980

[B26] Kapa L. L. Meyers-Denman C. Plante E. Doubleday K. (2020). Predictors of treatment response for preschool children with developmental language disorder. Am. J. Speech Lang. Pathol. 29, 2082–2096. 10.1044/2020_AJSLP-19-00198 32997549 PMC8740565

[B27] Kruythoff-Broekman A. Wiefferink C. Rieffe C. Uilenburg N. (2019). Parent-implemented early language intervention programme for late talkers: parental communicative behaviour change and child language outcomes at 3 and 4 years of age. Int. J. Lang. Commun. Disord. 54, 451–464. 10.1111/1460-6984.12451 30680870

[B28] Law J. Boyle J. Harris F. Harkness A. Nye C. (1998). Screening for speech language delay: a systematic review of the literature. Health Technol. Assess. 2, 1–184. 10.3310/hta2090 9728296

[B29] Law J. Garrett Z. Nye C. (2003). Speech and language therapy interventions for children with primary speech and language delay or disorder. Cochrane Database Syst. Rev. 3, CD004110. 10.1002/14651858.CD004110 12918003 PMC8407295

[B30] Law J. Garrett Z. Nye C. (2004). The efficacy of treatment for children with developmental speech and language delay/disorder: a meta-analysis. J. Speech Lang. Hear. Res. 47, 924–943. 10.1044/1092-4388(2004/069) 15324296

[B31] Lee W. Pring T. (2016). Supporting language in schools: evaluating an intervention for children with delayed language in the early school years. Child Lang. Teach. Ther. 32, 135–146. 10.1177/0265659015590426

[B32] Mouridsen S. E. Hauschild K. M. (2010). The sex ratio of siblings of individuals with a history of developmental language disorder. Logoped. Phoniatr. Vocol. 35, 144–148. 10.3109/14015430903518007 20735173

[B33] Ney C. Foster S. H. Seaman D. (1987). Effectiveness of language intervention with language/learning disabled children. J. Speech Hear. Res. 52, 348–357. 10.1044/jshd.5204.348 3669632

[B34] Roberts M. Y. Kaiser A. P. (2011). The effectiveness of parent-implemented language interventions: a meta-analysis. Am. J. Speech Lang. Pathol. 20, 180–199. 10.1044/1058-0360(2011/10-0055) 21478280

[B35] Roberts M. Y. Kaiser A. P. (2015). Early intervention for toddlers with language delays: a randomized controlled trial. Pediatrics 135. 686–693. 10.1542/peds.2014-2134 25733749 PMC4379460

[B36] Robertson S. B. Weismer S. E. (1999). Effects of treatment on linguistic and social skills in toddlers with delayed language development. J. Speech Lang. Hear. Res. 42, 1234–1248. 10.1044/jslhr.4205.1234 10515518

[B37] Shelton R. L. Johnson A. F. Ruscello D. M. Arndt W. B. (1978). Assessment of parent-administered listening training for preschool children with articulation deficits. J. Speech Hear. Dis. 43, 242–254. 10.1044/jshd.4302.242 661262

[B38] Smith-Lock K. Leitão S. Lambert L. Prior P. Dunn A. Cronje J. . (2013). Daily or weekly? The role of treatment frequency in the effectiveness of grammar treatment for children with specific language impairment. Int. J. Speech Lang. Pathol. 15, 255–267. 10.3109/17549507.2013.777851 23586584

[B39] Smith-Lock K. M. Leitão S. Prior P. Nickels L. (2015). The effectiveness of two grammar treatment procedures for children with SLI: a randomized clinical trial. Lang. Speech Hear. Serv. Sch. 46, 312–324. 10.1044/2015_LSHSS-14-0041 26110982

[B40] Sylvestre A. Desmarais C. Meyer F. Bairati I. Leblond J. (2018). Prediction of the outcome of children who had a language delay at age 2 when they are aged 4: still a challenge. Int. J. Speech Lang. Pathol. 20, 731–744. 10.1080/17549507.2017.1355411 28766373

[B41] Tamiya S. (2014). Multilingualism and child psychiatry: on differential diagnoses of language disorder, specific learning disorder, and selective mutism. Seishin Shinkeigaku Zasshi. 116, 907–920. 25702497

[B42] Tomblin J. B. Records N. L. Buckwalter P. Zhang X. Smith E. O'Brien M. (1997). Prevalence of specific language impairment in kindergarten children. J. Speech Hear. Res. 40, 1245–1260. 10.1044/jslhr.4006.1245 9430746 PMC5075245

[B43] Wake M. Tobin S. Levickis P. Gold L. Ukoumunne O. C. Zens N. . (2013). Randomized trial of a population-based, home-delivered intervention for preschool language delay preschool language delay. Pediatrics 132, e895. 10.1542/peds.2012-3878 24043276

[B44] Wang M. V. Aarø L. E Ystrom E. (2018). Language delay and externalizing problems in preschool age: a prospective cohort study. J. Abnorm. Child Psychol. 46, 923–933. 10.1007/s10802-017-0391-5 29322277

[B45] Wu P. L. Lee M. Huang T. T. (2017). Effectiveness of physical activity on patients with depression and Parkinson's disease: a systematic review. PLoS ONE 12, e0181515. 10.1371/journal.pone.0181515 28749970 PMC5531507

[B46] Xu G. Z. Jia J. Jin L. Li J. H. Wang Z. Y. Cao D. Y. (2018). Low-level laser therapy for temporomandibular disorders: a systematic review with meta-Analysis. Pain Res. Manag. 2018, 4230583. 10.1155/2018/4230583 29861802 PMC5971344

