# Supplementary material for: Effect of language therapy alone for developmental language disorder in children: A meta-analysis
Source: Front Psychol. 2022 Oct 3;13:922866. doi: 10.3389/fpsyg.2022.922866 (PMC9574219; doi:10.3389/fpsyg.2022.922866)
Supplement: Supplementary file 1 [file Table_1.docx]

Supplementary Table 1. Descriptive characteristics of the included 15 studies

| Study | Age | Number of participants | | Intervention type | Intervention duration | Language-specific assessment variables (assessment tools) |
| --- | --- | --- | --- | --- | --- | --- |
|  |  | Intervention | Control |  |  |  |
| Gibbard, 1994 | 2;3-3;3 (years; months) | 10 | 10 | Parent-based | 6 months | Expressive; comprehension (Reynell Developmental Language Scales);  Grammatical ability; information (Renfrew Action Picture Test);  One-word scores: expressive; total scores (Derbyshire Language Scheme Picture Test);  Word total; phrase length (Mother's description);  Mean length of utterance |
| Girolametto et al., 1996 | 22-33 months | 12 | 13 | Parent-based | 4 months | Expressive vocabulary; vocabulary size; Different target words; different control words (MacArthur Communicative Development Inventories (CDI));  Talkativeness/Rate; utterances; Words per minute (Systematic Analysis of Language Transcripts (SALT));  Structural complexity: Parent report on CDI (Number of grammatical structures used correctly on Part E of CDI-Words and Sentences); Multiword combinations |
| Girolametto et al., 1997 | 22-33 months | 12 | 13 | Parent-based | 4 months | Different vocalizations;  Syllable structure: Levels 1-3;  Consonant inventories: Early consonants/h, w j, m, n, d, p, b, f; Middle consonants/k, g, t, s, d; Late consonants/v, l, r, Q, D, tS, J, z, S;  Consonant position: Initial position; Final position;  Proportion of consonants correct |
| Almost and Rosenbaum, 1998 | 33-61 months | 13 | 13 | Therapist-based | 4 months (Test at 8 months) | Average of phonological deviations (APP-R);  Consonants correct in conversation speech (PCC);  Number of errors (GFTA);  Mean length of utterances in language sample |
| Robertson et al., 1999 | 21-30 months, (mean, 25.6 months) | 11 | 10 | Clinician-based | 12 weeks | Mean Length of Utterance; Total Number of Words; Number of Different Words (Systematic Analysis of Language Transcripts)  Lexical Repertoire (CDI);  Percent Intelligible Utterances (Inventory–Words and Sentences (CDI)) |
| Glogowska et al., 2000 | 34.5 (range 23 to 42) months | 71 | 88 | Therapist-based | 8.4 (0.9­12) months | Outcomes at 12 months follow-up:  Mean (range) auditory comprehension score;  Mean (range) expressive language score;  Mean phonology error rate (%)  Mean (SD) score on Bristol language development scale |
| Denne et al., 2005 | 5-7 years | 9 | 10 | Therapist-based | 8 weeks | Phonological Abilities Test;  Reading scores on Wechsler Objective Reading Dimension;  Spelling scores on Wechsler Objective Reading Dimension;  Scores on non-word reading;  Percentage of consonants correct scores on the South Tyneside Assessment of Phonology |
| Boyle et al., 2007 | 6-11 years | 130 | 31 | Nonparent-based | 15 weeks | Test at 12 months after the intervention:  Primary outcomes:  Receptive language (CELF-3^UK^-R);  Exceptive language (CELF-3^UK^-E);  Secondary outcomes:  Vocabulary (Vocabulary Scale–Second Edition (BPVS-II)) |
| Smith-Lock et al., 2013 | 5;1 (years; months) | 19 | 15 | Nonparent-based | 8 weeks | Grammar Elicitation Test |
| Wake et al., 2013 | 4 years | 93 | 91 | Nonparent-based | 10 months | Primary outcomes:  Expressive language; Receptive language (Clinical Evaluation of Language Fundamentals – Preschool, 2nd Edition (CELF-P2));  Secondary outcomes:  Pragmatic skills (Children’s Communication Checklist, 2nd Edition (CCC-2))  Letter knowledge (SPAT-R) |
| Buschmann et al., 2015 | 2 years | 23 | 20 | Therapist-guided | 6 months | Test at age of four  Phonological Abilities Test;  Reading scores on Wechsler Objective Reading Dimension;  Spelling scores on Wechsler Objective Reading Dimension;  Scores on non-word reading;  Percentage of consonants correct scores on the South Tyneside Assessment of Phonology |
| Roberts et al., 2015 | 24-42 months | 45 | 52 | Care-giver-guided | 3 months | Primary outcomes:  Expressive language; Receptive language (Preschool Language Scale, Fourth Edition);  Secondary outcomes:  Expressive vocabulary (EOWPVT-3);  Expressive vocabulary (NDW);  Expressive vocabulary (MCDI) |
| Lee and Pring, 2016 | 4-7 years | 62 | 53 | Nonparent-based | 10 weeks | Receptive language; Receptive grammar (Renfrew Action picture test);  Receptive language (Language use assessed by Bus Story (Renfrew, 1997) |
| Dawes et al., 2019 | 5-6 years | 19 | 18 | Nonparent-based | 8 weeks | Test at 2 months after intervention:  Expressive vocabulary (Active Vocabulary Test for 3- to 5-Year-Old Children);  Sentence comprehension (SETK) |
| Hampton et al., 2017 | 24-42 months | 45 | 43 | Parent-based | 3 months | After intervention:  Primary outcomes:  Expressive; receptive (PLS-4);  Secondary outcomes:  Number of total utterances;  Number of different word roots in a 20-minute play interaction;  Macarthur-Bates Communication Development Inventories;  Expressive One-Word Picture Vocabulary Test;  Peabody Picture Vocabulary Test |
|  |  | 42 | 38 |  |  | 12 months after the completion of the intervention:  Primary outcomes:  Expressive; receptive (PLS-4);  Secondary outcomes:  Number of total utterances;  Number of different word roots in a 20-minute play interaction;  Macarthur-Bates Communication Development Inventories;  Expressive One-Word Picture Vocabulary Test;  Peabody Picture Vocabulary Test |
| Ebbels et al., 2012 | 9;11–15;11 (years; months) | 8 | 7 | Nonparent-guided | 8 weeks | Phase 1 (immediate after the intervention):  Word finding (TAWF RS; TAWF Prorated SS);  Semantic fluency (PhAB semantic fluency SS);  Percentage T-units with > 0 (word finding difficulties) WFDs;  WFDs/T-unit;  Percentage (WFDs/total words);  Mean of three word categories;  Phase 2 (five months after phase-1 intervention; self-controlled)  Word finding (TAWF RS; TAWF Prorated SS) |
